# ADAR1 enhances HTLV-1 and HTLV-2 replication through inhibition of PKR activity

**DOI:** 10.1186/s12977-014-0093-9

**Published:** 2014-11-12

**Authors:** Anne Cachat, Sandrine Alais, Sébastien Alain Chevalier, Chloé Journo, Floriane Fusil, Hélène Dutartre, Adrien Boniface, Nga Ling Ko, Antoine Gessain, François-Loïc Cosset, Rodolphe Suspène, Jean-Pierre Vartanian, Renaud Mahieux

**Affiliations:** Equipe Oncogenèse Rétrovirale, Lyon, Cedex 07 France; Equipe labellisée “Ligue Nationale Contre le Cancer”, Lyon, Cedex 07 France; Centre international de recherche en infectiologie, INSERM U1111 - CNRS UMR5308, Lyon, Cedex 07 France; Ecole Normale Supérieure de Lyon, 46 allée d’Italie, 69364 Lyon, Cedex 07 France; Université Lyon 1, LabEx ECOFECT - Eco-evolutionary dynamics of infectious diseases, 69364 Lyon, Cedex 07 France; Equipe virus enveloppés, Lyon, Cedex 07 France; Biology Department, Master Biosciences, Lyon, Cedex 07 France; Unité d’épidémiologie et physiopathoglogie des virus oncogènes, Institut Pasteur, Paris, 75015 France; Unité de rétrovirologie moléculaire, Institut Pasteur, Paris, 75015 France

**Keywords:** HTLV, Interferon, ADAR1, PKR

## Abstract

**Background:**

The role of innate immunity in general and of type I interferon (IFN-I) in particular in HTLV-1 pathogenesis is still a matter of debate. ADAR1-p150 is an Interferon Stimulated Gene (ISG) induced by IFN-I that can edit viral RNAs. We therefore investigated whether it could play the role of an anti-HTLV factor.

**Results:**

We demonstrate here that ADAR1 is also expressed in the absence of IFN stimulation in activated primary T-lymphocytes that are the natural target of this virus and in HTLV-1 or HTLV-2 chronically infected T-cells. ADAR1 expression is also increased in primary lymphocytes obtained from HTLV-1 infected individuals. We show that ADAR1 enhances HTLV-1 and HTLV-2 infection in T-lymphocytes and that this proviral effect is independent from its editing activity. ADAR1 expression suppresses IFN-α inhibitory effect on HTLV-1 and HTLV-2 and acts through the repression of PKR phosphorylation.

**Discussion:**

This study demonstrates that two interferon stimulated genes, i.e. PKR and ADAR1 have opposite effects on HTLV replication *in vivo*. The balanced expression of those proteins could determine the fate of the viral cycle in the course of infection.

**Electronic supplementary material:**

The online version of this article (doi:10.1186/s12977-014-0093-9) contains supplementary material, which is available to authorized users.

## Background

Human T-cell leukemia virus type 1 (HTLV-1) was the first human oncoretrovirus to be discovered [1,2]. It infects 5 to 10 million people worldwide [3]. Among them, 3 to 5 percent have a lifetime risk to develop a lymphoproliferative disorder named adult T-cell leukemia (ATL) [4], or an inflammatory neurodegenerative disease named HTLV-1 associated myelopathy/tropical spastic paraparesis (HAM/TSP) [5,6]. HTLV-2 infects 2 to 5 million people. Despite similarities with HTLV-1 in its genomic organization, HTLV-2 is only associated with rare cases of HAM/TSP-like diseases but does not promote leukemia or lymphoma [7,8]. The cause for such differences is a matter of investigation [9-18]. Nevertheless multiple reports already pointed out the key roles played both by Tax and HTLV-1 antisense-encoded protein in cell transformation [18-20].

Innate immunity plays a critical role in host response to a viral infection by triggering effector mechanisms that restrict infection [21]. Effectors of innate immunity such as restriction factors are present in cells before infection takes place and can act as immediate inhibitors of a given infectious agent. Double stranded RNA which is produced during replication of several viruses is a potent inducer of IFN-I which then acts as a signal to induce expression of antiviral effectors, among which Adenosine Deaminase Acting on RNA (ADAR), a cellular protein which edits RNA [22]. RNA editing which does not include capping, 3’ processing, and splicing is defined as post-transcriptional events that modify RNA molecules. It is a natural process used by mammal cells to generate different variants of a given protein. ADAR enzymes are capable of editing double stranded RNA ([23] and for a recent review see [24]). There are three members of the mammalian ADAR family: ADAR1, ADAR2 and ADAR3. The formers convert adenosines into inosines, which are then translated into guanosines, while ADAR3 has no catalytic activity and seems mainly restricted to nervous system [25,26].

There are two ADAR1 isoforms. ADAR1-p110 is constitutively expressed and found exclusively in the nucleus, while ADAR1-p150 is an Interferon Stimulated Gene (ISG) induced by IFN-I [27]. ADAR1-p150 is found in the nucleus and in the cytoplasm [28,29]. These two isoforms are generated from two distinct promoters and an alternative splicing of the first exon. ADAR1-p110 and ADAR1-p150 have a C-terminal catalytic deaminase domain, three dsRNA binding domains and one Z-DNA binding domain in ADAR1-p110 or two in ADAR1-p150. Both ADAR1 isoforms edit cellular and viral RNAs possessing a double-stranded structure [30,31]. Interestingly, several viruses produce dsRNA during their replication cycle [22].

As ADAR1-p150 expression can be induced by type I interferon, it was anticipated that it would be an effector of type I interferon antiviral effect on viral replication. Indeed, viral genome editing by ADAR1 was described for several viruses [30,32]. For example, ADAR1 edits hepatitis C viral genome and consequently reduces the level of HCV subgenomic RNA [33]. ADAR1 possesses a repressive activity against lymphocytic choriomeningitis virus (LCMV) [34], and bovine viral diarrhea virus (BVDV) [35]. Surprisingly however, ADAR1 plays a proviral role for other viruses such as vesicular stomatitis virus [36,37], Kaposi sarcoma-associated herpesvirus [38], Epstein-Barr virus [39], dengue virus [40] and Rift valley virus [31], while controversial results on hepatitis D, measles and influenza replication have been published [31,40-49]. ADAR1-p110 and ADAR1-p150 expression increases during the course of infection and promotes HIV-1 viral replication, thus playing a proviral role [50-53]. However, in this case, conflicting mechanisms have also been suggested: one study suggested that this effect requires ADAR1 editing activity, while another showed that ADAR1 proviral effect is independent from its catalytic site, but relies on the inhibition of the protein kinase R (PKR) function, which normally inhibits mRNA translation and induces apoptosis [50-52]. ADAR1-mediated PKR inhibition was also reported during measles virus or vesicular stomatitis virus infection [36,37,49]. Thus, depending on the viruses, ADARs can act either as antiviral or as proviral factors (for recent reviews, see [30,32]).

IFN-α is being used in combination to AZT to treat ATL patients, although the relationship between stimulation of innate immunity and impact on HTLV cycle are still not fully understood [21,54,55]. We recently demonstrated that PKR prevented HTLV-1 and -2 replication in cell culture following IFN-I treatment [11]. In another set of experiments using the exquisitely sensitive 3DI-PCR technique [56], we have also shown that HTLV-2 and simian T-cell leukemia virus type 3 (STLV-3) genomes can be edited by ADAR1 in cells transfected with HTLV-2 or STLV-3 molecular clones and an ADAR1-encoding plasmid [57]. However we could not find such mutations in *ex vivo* samples obtained from HTLV-2 infected individuals, thus demonstrating that ADAR1 editing of HTLV *in vivo* is a very rare phenomenon and is unlikely to play an antiviral role [57]. Though, consequences of ADAR1 expression on HTLV replication were not fully investigated in this report.

Here, we first demonstrate that ADAR1 is expressed in PHA/IL-2 activated primary T-lymphocytes in the absence of IFN-I stimulation, in HTLV-1 or HTLV-2 chronically infected T-cells and in primary CD4^+^ cells obtained from HTLV-1 infected individuals. We show that ADAR1 enhances HTLV-1 and HTLV-2 infection in T-lymphocytes and that this proviral effect is independent from its editing activity. ADAR1 expression suppresses IFN-α inhibitory effect on HTLV-1 and HTLV-2 through repression of PKR phosphorylation. Together with our previous results, these studies demonstrate that two interferon stimulated genes, i.e. PKR and ADAR1 have opposite effects on HTLV replication *in vivo*. The balanced expression of those proteins could determine the fate of viral cycle in the course of infection.

## Results

### ADAR1 is expressed in activated peripheral blood lymphocytes and in HTLV-1 and HTLV-2 chronically infected T cell lines

**Figure 1:**
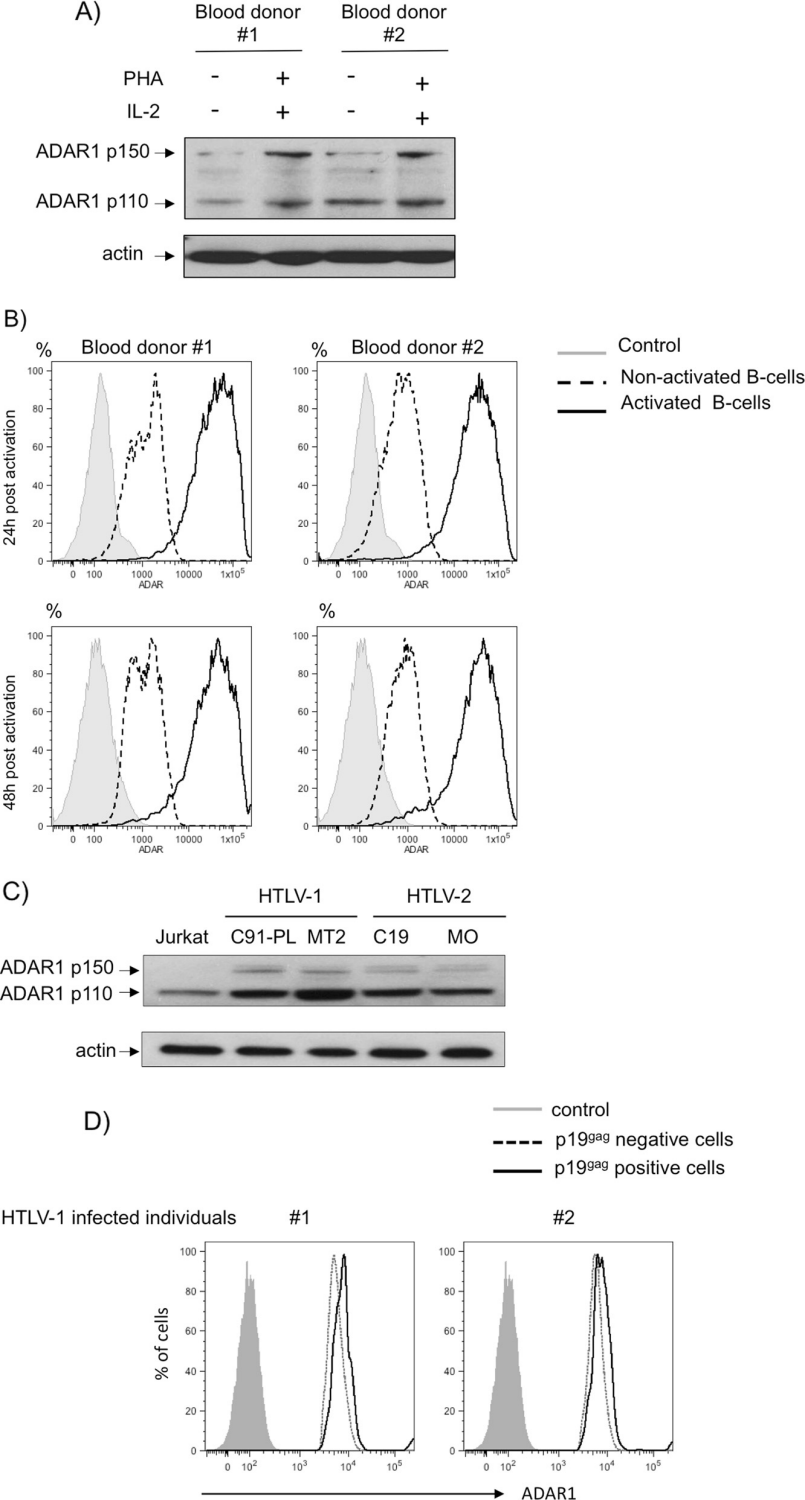
**ADAR1 is expressed in activated lymphocytes as well as in HTLV-1 and HTLV-2 chronically infected T-cell lines and primary T-lymphocytes from HTLV-1 infected individuals. (A)**: Whole cell extracts (60 μg) obtained from PBLs activated or not with PHA/IL-2 for 72 hours. **(B)**: B-cells were purified from the PBMCs of two blood donors and left unactivated (dot line) or treated with Pansorbin (solid line). ADAR1 expression was measured 24 h and 48 h later (FACS Canto II, BD Biosciences). Isotype control is represented with a grey histogram **(C)**: Uninfected- (Jurkat), HTLV-1-infected (C91-PL, MT2) and HTLV-2-infected (C19, MO) cells were analyzed by western-blot analyses using anti-ADAR1 or anti-actin antibody. **(D)**: CD4^+^ T-cells from two HTLV-1 positive individuals were sorted, stimulated with PHA and cultured in the presence of IL-2. Cells were stained with anti ADAR1 and anti HTLV p19^gag^ antibodies followed by APC anti-rabbit and FITC anti-mouse antibodies. CD4^+^ cells from p19^gag^ negative population (dot line) or from p19^gag^ positive population (solid line) were analyzed for ADAR1 expression. **(B, D)**: Cells were analyzed on a FACS-Canto II (BDSciences) collecting 100 000 events. Results analyzed using FlowJo software.

ADAR1 was previously shown to edit the genome of some RNA viruses and to act therefore as an antiviral factor [31,34,44,45]. Consequently we first wondered whether ADAR1 was expressed in primary uninfected T-lymphocytes that are the target of HTLV-1 and -2 retroviruses *in vivo*. Given that HTLV-1 infected T-lymphocytes display an activated phenotype [58], we first obtained Peripherical Blood Lymphocytes (PBLs) from two healthy blood donors and activated them with PHA and IL-2. An aliquot of the same cells was also left quiescent. As expected, ADAR-p110 form was constitutively expressed, while ADAR1-p150 expression was induced following T-cell activation (Figure 1A). As a control, primary B-cells were also purified from normal PBLs and left quiescent or activated with Pansorbin for 24 and 48 hours. As observed for T-cells, B-cell activation led to a strong increase in ADAR-1 expression (Figure 1B), thus suggesting that increase in ADAR1 expression is linked to cell activation.

Then, we compared the expression of both ADAR1 isoforms in non-infected Jurkat T-lymphocytes as well as in HTLV-1 (C91-PL and MT2) or HTLV-2 (C19 and MO) infected T-lymphocytes (Figure 1C). While ADAR1-p110 was expressed in all transformed cells including Jurkat, ADAR1-p150 was specifically up-regulated in HTLV-1 and HTLV-2 chronically infected cells.

Finally, CD4^+^ T-cells were isolated from the blood of two HTLV-1 infected individuals and stimulated with PHA and IL-2 for 3 days then grown only with IL-2. For each sample, ADAR-1 expression was then measured by flow cytometry in the fraction of CD4^+^ cells that express HTLV-1 (p19^gag^) antigen and compared to the p19^gag^ negative population. Interestingly, HTLV-1 productively infected cells expressed higher levels of ADAR1 than cells that were also stimulated but did not express HTLV-1 antigens (Figure 1D).

Altogether, these results show that both isoforms of ADAR1 are expressed in the absence of IFN-I stimulation in activated T-cells that are susceptible to HTLV-1 and HTLV-2 infection. They also demonstrate that HTLV-1 and HTLV-2 chronically infected cells express the inducible ADAR1 isoform and suggest that HTLV-1 expression is correlated with an increased ADAR1 expression in primary CD4^+^ T-cells.

### ADAR1 has a proviral effect on *de novo* HTLV-1 and HTLV-2 infection in T- lymphocytes

**Figure 2:**
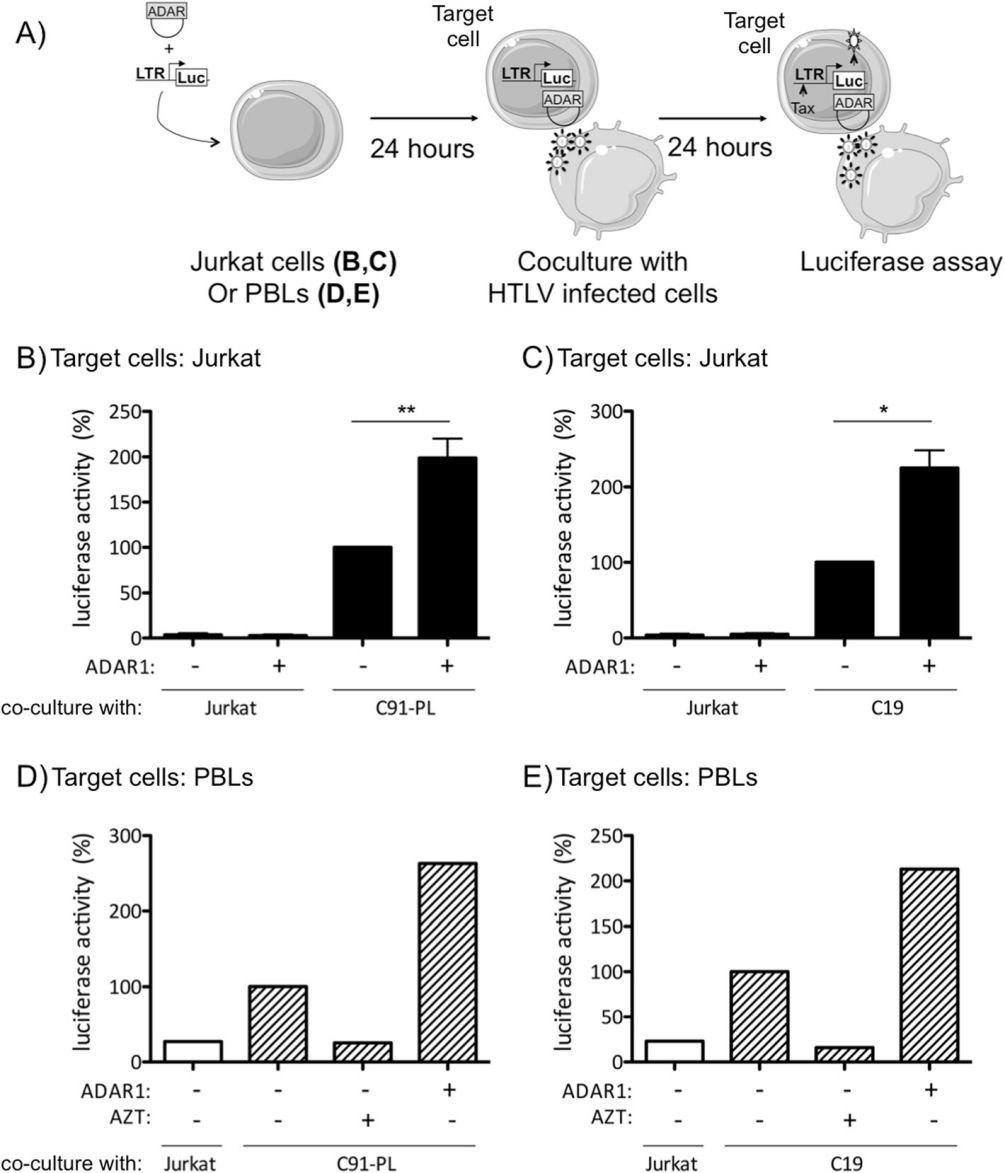
**ADAR1 has a proviral effect on** ***de novo*** **HTLV-1 and HTLV-2 infection in T-lymphocytes. (A)**: Jurkat or PBLs were transfected either with 2.5 μg of an ADAR1 or of a backbone vector, together with 2.5 μg of a plasmid encoding the luciferase gene under the control of the HTLV-1-LTR or HTLV-2-LTR. Twenty-four hours later, Jurkat **(B, C)** or PBLs **(D, E)** were co-cultured with **(B, D)** irradiated C91-PL (HTLV-1), **(C, E)** C19 (HTLV-2) or **(B, C, D, E)** Jurkat (non-infected) cells for 24 hours. Luciferase activity was measured, and results normalized by protein concentration as determined by the Bradford method and calculated as fold change compared to infected cells transfected with the control plasmid arbitrarily set to 100%. **(D, E)**: PBLs were treated with AZT (50 μM) before the co-culture with C91-PL or C19 irradiated cells. **(B, C)**: Data are the mean ± standard deviation (SD) from 3 independent experiments. Asterisks indicate statistically significant differences between treated and untreated cells (paired Student *t* test, *p < 0.05; **p < 0.01). **(D, E)**: Data are representative of two different experiments obtained with two different blood donors.

Then, we investigated the effect of ADAR1 expression on *de novo* infection of T-cells with HTLV-1 and HTLV-2 using a previously described protocol (Figure 2A) [11].

HTLV-1/-2 infection occurs mainly through cell-cell contact. Target Jurkat (Figure 2B, C) or primary PBLs (Figure 2D, E) were therefore co-transfected with a plasmid encoding ADAR1 and a plasmid encoding the luciferase indicator gene under the control of the HTLV-1 or HTLV-2 LTR promoters. Twenty-four hours later, target cells were co-cocultured with gamma-irradiated C91-PL (HTLV-1), C19 (HTLV-2) donor cells or non-infected Jurkat cells. The viral non-structural Tax protein is necessary for activating transcription from the LTR. Consequently, luciferase activity monitors viral infection i.e. viral entry, reverse transcription, integration and viral expression in target cells (Figure 2A).

ADAR1 expression in target Jurkat cells significantly enhanced luciferase activity after co-culture with HTLV-1 or HTLV-2 infected irradiated T-cells (Figure 2B compare lane 3 vs. 4, and Figure 2C lane 3 vs. 4). These results suggest that ADAR1 increases target cells susceptibility to HTLV-1 and HTLV-2 infection. As a control, ADAR1 did not affect the level of luciferase activity in target Jurkat target cells co-cultured with non-infected T-cells (Figure 2B and C lanes 1-2).

Similar experiments were then performed using primary PBLs obtained from healthy blood donors as target cells, instead of Jurkat cells. As in Jurkat, ADAR1 expression in PBLs was associated with an increased luciferase activity (Figures 2D and E compare lane 2 vs. 4). To control that luciferase activity was linked to viral infection and not a passive Tax transfer from donor to target cells, we performed a series of control experiments where PBLs were treated or not with AZT, an inhibitor of reverse transcription. As expected, AZT treatment strongly reduced luciferase activity thus demonstrating that *de novo* Tax synthesis is required in the target cells (Figure 2D and E, lanes 2 vs.3).

These results demonstrate that ADAR1 has a proviral effect on HTLV-1 and HTLV-2 *de novo* infection both in T-cell lines and in primary T- lymphocytes.

### ADAR1 increases HTLV-1 and HTLV-2 protein expression

**Figure 3:**
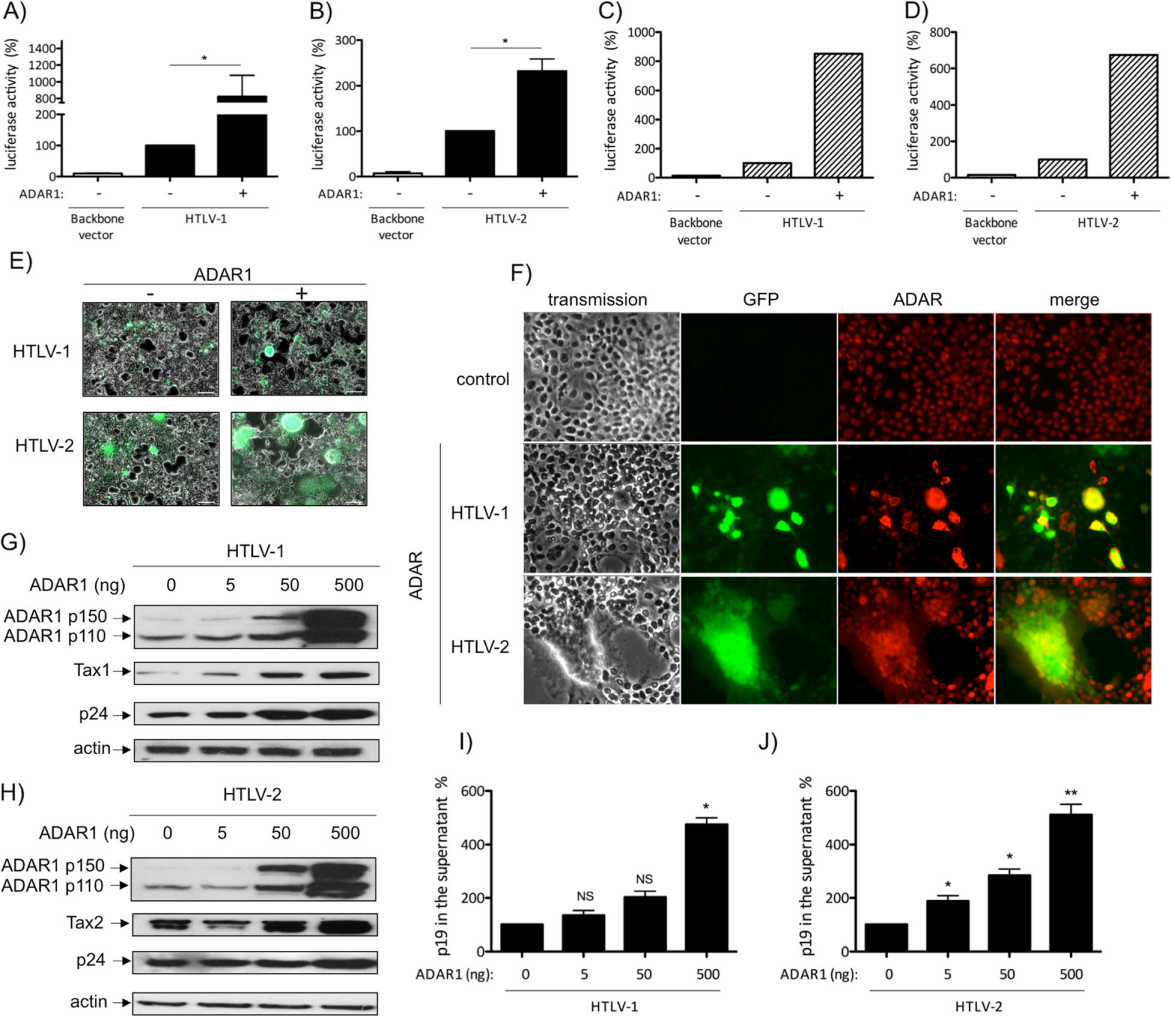
**ADAR1 expression enhances HTLV-1 and HTLV-2 protein expression. (A, B)**: Jurkat or **(C, D)** PBLs were transfected with **(A, C)** 2 μg of pACH or **(B, D)** pH6neo molecular clones together with 1 μg of a plasmid encoding ADAR1 and 2 μg of **(A, C)** HTLV-1-LTR or **(B, D)** HTLV-2-LTR-luc plasmids. Forty-eight hours later, luciferase activity was measured and results normalized by protein concentration as determined by the Bradford method and calculated as fold change compared to cells that were not transfected with ADAR1 plasmid arbitrarily set to 100%. **(A, B)**: Data are the mean ± standard deviation (SD) from 3 independent experiments. Asterisks indicate statistically significant differences between treated and untreated cells (paired Student *t* test, *p < 0.05). **(C, D)**: Data are representative of two different experiments obtained with two different blood donors. **(E, F)**: 293T-LTR-GFP cells were transfected with 1 μg of pACH (HTLV-1) or pH6neo (HTLV-2) molecular clones together with 125 ng of an ADAR1 expression plasmid or of the backbone vector. Forty-eight hours later, cells were visualized and pictures taken with a Zeiss Axio Vert.A1 imaging microscope. **(G, H, I, J)**: 293 T cells were transfected with 4 μg of **(G, I)** pACH (HTLV-1) or **(H, J)** pH6neo (HTLV-2) molecular clones and increasing amount (0, 5, 50, 500 ng) of an ADAR1 expression plasmid. **(G, H)**: Western blot analyses were performed on 60 μg of proteins obtained from whole cell extracts using anti-ADAR1, anti-p24^gag^, anti-Tax1, anti-Tax2 and anti-actin antibodies. **(I, J)**: p19^gag^ was quantified in the culture supernatants from cells transfected as in **(G, H)**. Data are represented as fold change compared to the p19^gag^ values obtained for cells that were not transfected with the ADAR1 plasmid arbitrarily set to 100%.

To decipher how ADAR1 affects HTLV-1/2 replication, Jurkat (Figure 3A-B) or primary T-lymphocytes (Figure 3C-D) were transfected with molecular clones which contain full length proviruses together with HTLV-1 or HTLV-2-LTR-luciferase reporter, in the presence or absence of ADAR1. This system allows us to bypass the entry-integration viral steps cells. In both settings, ADAR1 expression was associated with an increased luciferase activity, thus indicating up-regulation of Tax1 and Tax2 protein expression (Figure 3A-D compare lane 2 and 3). Of note, in the presence of ADAR1 plasmid, a higher increase in luciferase activity was observed when Jurkat cells were transfected with the HTLV-1 molecular clone than with the HTLV-2 clone. This effect was not observed in primary T-cells.

293T-LTR-GFP cells were then transfected with HTLV-1 or HTLV-2 molecular clones. These cells stably harbor a GFP reporter gene whose expression parallels activation of LTR-driven transcription. Consistent with co-culture experiments, ADAR1 expression increased the number of GFP positive cells, thus demonstrating an increased Tax-mediated LTR transcription (Figure 3E compare minus and plus ADAR1). ADAR1 expression was also associated with an increased number of syncitia, indicative of a higher HTLV envelope protein expression (Figure 3E, compare minus and plus ADAR1). In addition, most GFP positive cells were also strongly positive for ADAR (Figure 3F). Western-blot analyses, performed on 293T-LTR-GFP cells transfected with a constant amount of HTLV-1 (Figure 3G) or HTLV-2 molecular clone (Figure 3H) and increasing amounts of ADAR1 confirmed those observations. Indeed, both Tax1, Tax2 and p24^gag^ protein expression levels increased in a dose dependent manner (Figure 3G-H). As a control, actin levels remain constant under the same conditions. The amount of p19^gag^ in the culture supernatant, which is assumed to reflect the quantity of viral particles, was also measured in the same experimental conditions. Consistent with western-blot results, p19^gag^ in the supernatant also increased in a dose-dependent manner (Figure 3I, J).

Thus, these results demonstrate that ADAR1 increases viral expression and viral production in all cell lines tested.

### ADAR1 expression does not impair the production of infectious viral particles

**Figure 4:**
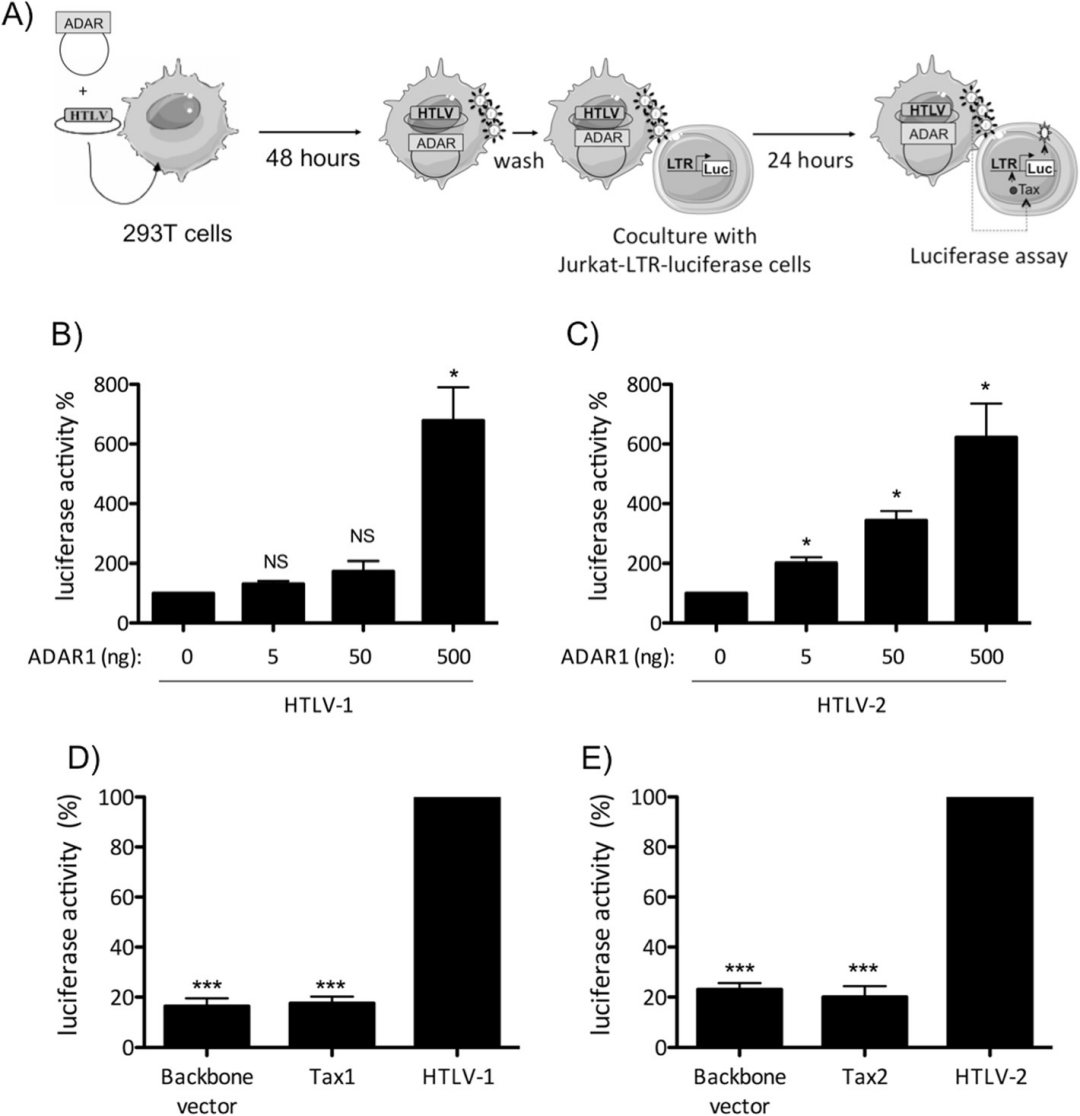
**ADAR1 expression does not affect viral infectivity. (A)**: 293-T cells were transfected with 4 μg of **(B)** pACH or **(C)** pH6neo molecular clones and increasing amount (0, 5, 50, 500 ng) of an ADAR1 expression plasmid or 8 μg of **(D)** pSG5M-Tax1, **(E)** pSG5M-Tax2 or backbone vector. Forty-eight hours later, cells were washed and co-cultured with Jurkat-LTR-luc cells for 24 hours (ratio 1:3). Luciferase activity results were normalized to protein concentration as determined by the Bradford method and calculated as fold change compared to cells non transfected with ADAR1 plasmid arbitrarily set to 100%. Data are the mean ± standard deviation (SD) from 3 independent experiments. Asterisks indicate statistically significant differences between treated and untreated cells (paired Student *t* test, *p < 0.05; ***p < 0.001; NS: non significant).

Given that ADAR1 possesses an editing activity, we then wondered whether viral particles produced in the presence of ADAR1 were infectious or not. 293T cells were transfected with HTLV-1 or HTLV-2 molecular clones and increasing amount of ADAR1 plasmid. Forty-eight hours after transfection culture medium was removed, cells were extensively washed before being co-cultured with Jurkat-LTR-luciferase cells (Figure 4A). We observed a dose-dependent increase in luciferase activity, suggesting that viral particles that are made in the presence of ADAR1 are still capable of replication in their target cells (Figure 4B, C). To control that luciferase activity measured in Jurkat target cells was not due to a passive Tax transfer from the transfected cell to the target cell, 293-T cells were transfected with Tax1 or Tax2 plasmids and co-cultured with Jurkat-LTR-luciferase cells. Luciferase activity was measured and was similar to that of 293T cells transfected with a backbone vector (Figure 4D, E).

Altogether, these results demonstrate that ADAR1 has a proviral effect, enhances HTLV-1/-2 expression and release of infectious particles.

### ADAR1 proviral effect is independent from its editing activity

We next wondered whether ADAR1 proviral effect was dependent or not of its editing activity. Using a previously described ADAR1 RNA editing reporter system [40], we first verified that the plasmid encoding ADAR1 with a mutation in its catalytic site was deficient for its editing activity. The unedited reporter plasmid contains a stop codon that is converted into Trp codon when A to I editing by ADAR1 occurs. As a consequence, editing by ADAR1 is correlated with Firefly luciferase expression. As expected, transfection of wild-type ADAR1 induced an increased luciferase activity (Additional file 1: Figure S1 left graph), while ADAR1 mutant did not lead to any increase in luciferase expression (Additional file 1: Figure S1 right graph).

**Figure 5:**
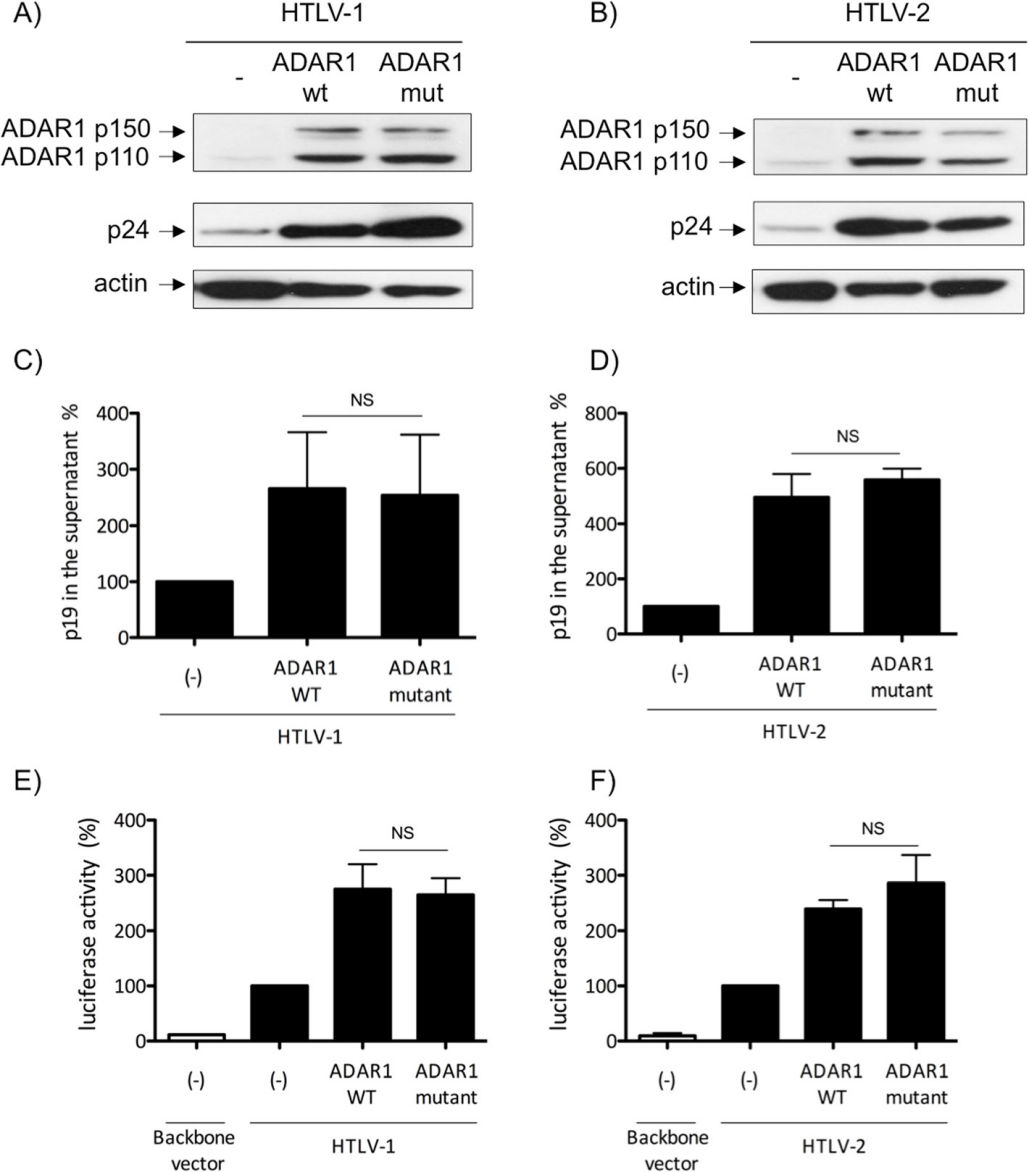
**ADAR1 enhances HTLV-1 and HTLV-2 replication independently from its editing activity.** 293-T cells were transfected with **(A, C)** 4 μg of pACH or **(B, D)** pH6neo molecular clones together with 500 ng of a plasmid coding for ADAR1 wild-type or for an editing-deficient ADAR1 protein (ADAR1 mutant). **(A, B)**: Western blot analyses were performed on 60 μg of proteins obtained from whole cell extracts using anti-p24^gag^, anti-ADAR1 and anti-actin antibodies. **(C, D)**: p19^gag^ was quantified in supernatant obtained from cells transfected as in **(A, B)**. Data are represented as fold change compared to the p19^gag^ values obtained for cells that were not transfected with the ADAR1 plasmid arbitrarily set to 100%. **(E, F)**: Jurkat cells were transfected with 2 μg of **(E)** pACH or **(F)** pH6neo molecular clones, together with 1 μg of a plasmid encoding either ADAR1 wild-type or ADAR1 mutant and 2 μg of **(E)** HTLV-1-LTR or **(F)** HTLV-2-LTR-luc plasmid. Forty-eight hours later, luciferase activity was measured and results normalized to protein concentration as determined by the Bradford method and calculated as fold change compared to cells non transfected with ADAR1 plasmid arbitrarily set to 100%. **(C-F)**: Data are the mean ± standard deviation (SD) from 3 independent experiments. Asterisks indicate statistically significant differences between treated and untreated cells (paired Student *t* test; NS: non significant).

Then, 293-T cells were transfected with HTLV-1 or HTLV-2 molecular clones, in the presence of plasmids encoding either wild-type ADAR1 or the editing-deficient ADAR1 mutant. p24^gag^ western-blot (Figure 5A, B) or p19^gag^ ELISA (Figure 5C, D) revealed that expression of both wild-type or mutant ADAR1 led to a similar increase in viral protein expression and virus release.

Next, to confirm that editing has no effect on HTLV transcription, we used Jurkat cells transfected with the HTLV-LTR-luciferase reporter plasmids and co-transfected with HTLV-1 or HTLV-2 molecular clone, together with wild-type or editing-deficient ADAR1 constructs (Figure 5E, F). A similar increase in luciferase activity was observed with wild-type or mutant ADAR1. We also determined whether viral particles produced in the presence of both wild-type or mutant ADAR1 plasmids were infectious by performing a coculture assay as described in Figure 2A. ADAR1 mutant expression increased the release of infectious viral particles similarly to ADAR1 wild-type both in HTLV-1 and HTLV-2 settings (Additional file 2: Figure S2).

Altogether, these results confirm that ADAR1 proviral effect on HTLV-1 and HTLV-2 replication is independent from its editing activity.

### ADAR1 proviral effect is mediated through inhibition of PKR activation

**Figure 6:**
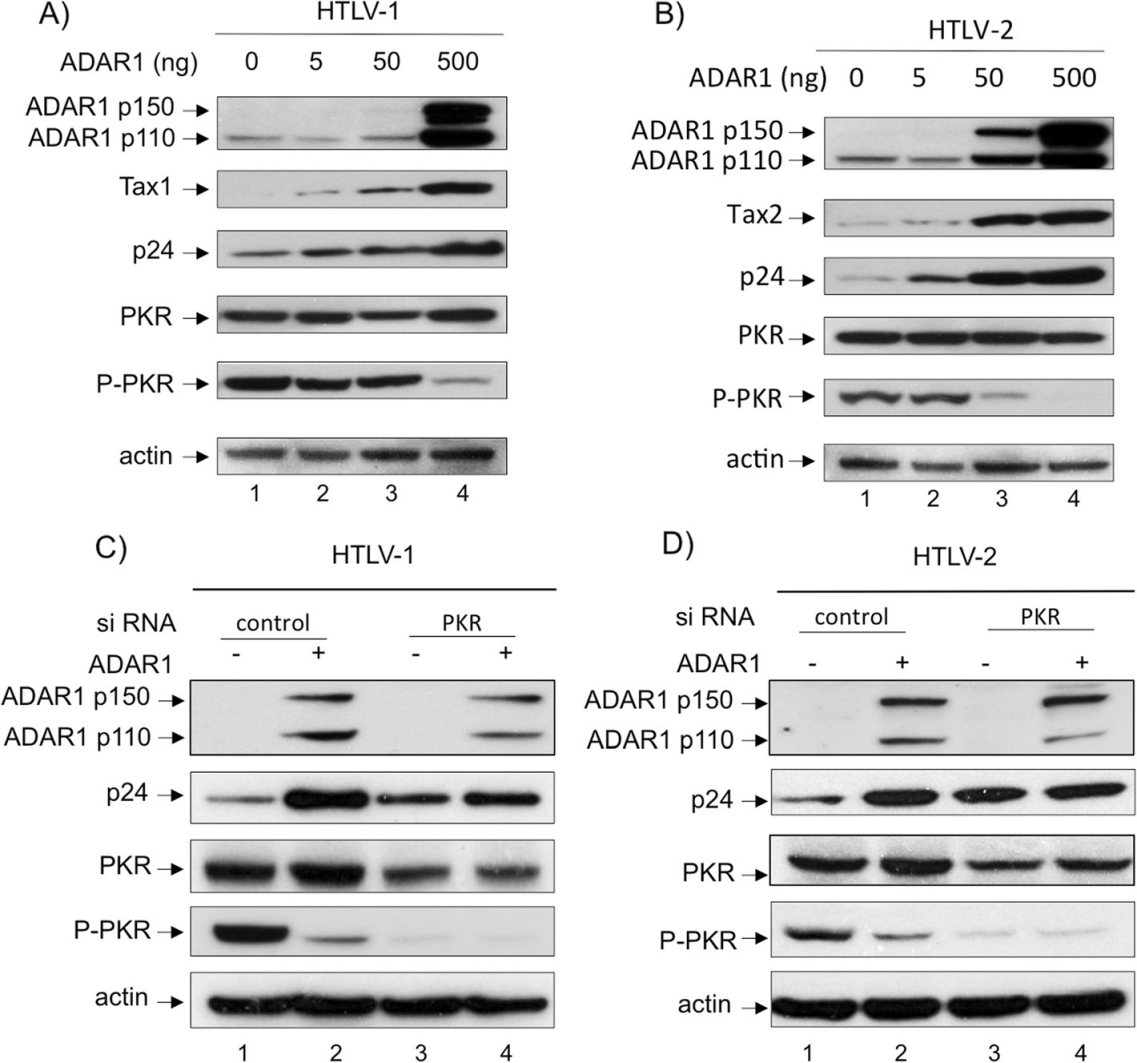
**ADAR1 proviral effect is mediated through inhibition of PKR activation.** 293-T cells were transfected with 4 μg of **(A)** pACH or **(B)** pH6neo molecular clones and increasing amount (0, 5, 50, 500 ng) of ADAR1 plasmid. Western blot analyses were performed on 60 μg of proteins obtained from whole cell extracts using anti-ADAR1, anti-p24^gag^, anti-Tax1, anti-Tax2, anti-PKR, anti-phospho-PKR and anti-actin antibodies. **(C, D)**: 293-T cells were transfected with siRNA directed against PKR (20nM) or with control siRNA. Twelve hours later cells were transfected with 20nM of the same siRNA together with 1.2 μg of **(C)** pACH, **(D)** pH6neo and 125 ng of an ADAR1 expression plasmid. Western blot analyses were performed on 60 μg of proteins obtained from whole cell extracts using anti-ADAR1, anti-p24^gag^, anti-PKR, anti-phospho-PKR and anti-actin antibodies.

PKR is an ISG that has been shown to be involved, once activated by phosphorylation, in a signaling pathway leading to inhibition of mRNA translation. It was previously established that ADAR1 antagonizes PKR activation [37,49]. This effect requires double-strand RNA binding of ADAR [37]. We therefore wondered whether ADAR1 proviral effect on HTLV-1 and HTLV-2 could be mediated through PKR inhibition. To test this hypothesis, PKR western-blots were performed on protein extracts obtained from 293-T cells transfected with HTLV-1 and HTLV-2 molecular clones together with increasing amount of ADAR1 (Figure 6A, B). Consistent with results presented above, the amount of p24^gag^ and Tax viral proteins increased in an ADAR1 dose dependent manner, while the level of PKR remained constant (Figure 6A, B compare lane 1 vs 2-4). Interestingly, the level of phospho-PKR (i.e. the active form of PKR) was inversely correlated with that of ADAR1 (Figure 6A, B, compare lane 1 and 4).

To demonstrate that ADAR1 proviral effect was linked to the inhibition of PKR activation, cells were transfected either with PKR siRNA or with control siRNA before being transfected with HTLV-1/2 molecular clones and ADAR1 plasmid (Figure 6C, D). As previously described [11], PKR extinction results in an increased p24 expression. However, despite ADAR1 ectopic expression, the level of p24^gag^ remained constant, in cells transfected with PKR siRNA, while it increased when control siRNA were transfected.

These results demonstrate that ADAR1 effect on HTLV-1 and HTLV-2 is linked to the inhibition of PKR phosphorylation. A previous study demonstrated that HIV-1 Tat interacts with PKR and inhibited PKR auto-phosphorylation thus providing a possible mechanism of IFN suppression [59]. We therefore tested whether Tax could also bind and inhibit PKR. However no interaction between Tax and PKR was observed (data not shown).

### ADAR1 expression reverses IFN-α inhibitory effect on HTLV-1 and HTLV-2

We have recently shown that IFN-α restricts HTLV-1 and HTLV-2 *de novo* infection by inhibiting viral protein synthesis through PKR activation [11]. Since ADAR1 inhibits PKR activation and enhances viral protein translation, we finally wondered whether IFN-α still had an antiviral effect in the presence of ADAR1 ectopic expression.

**Figure 7:**
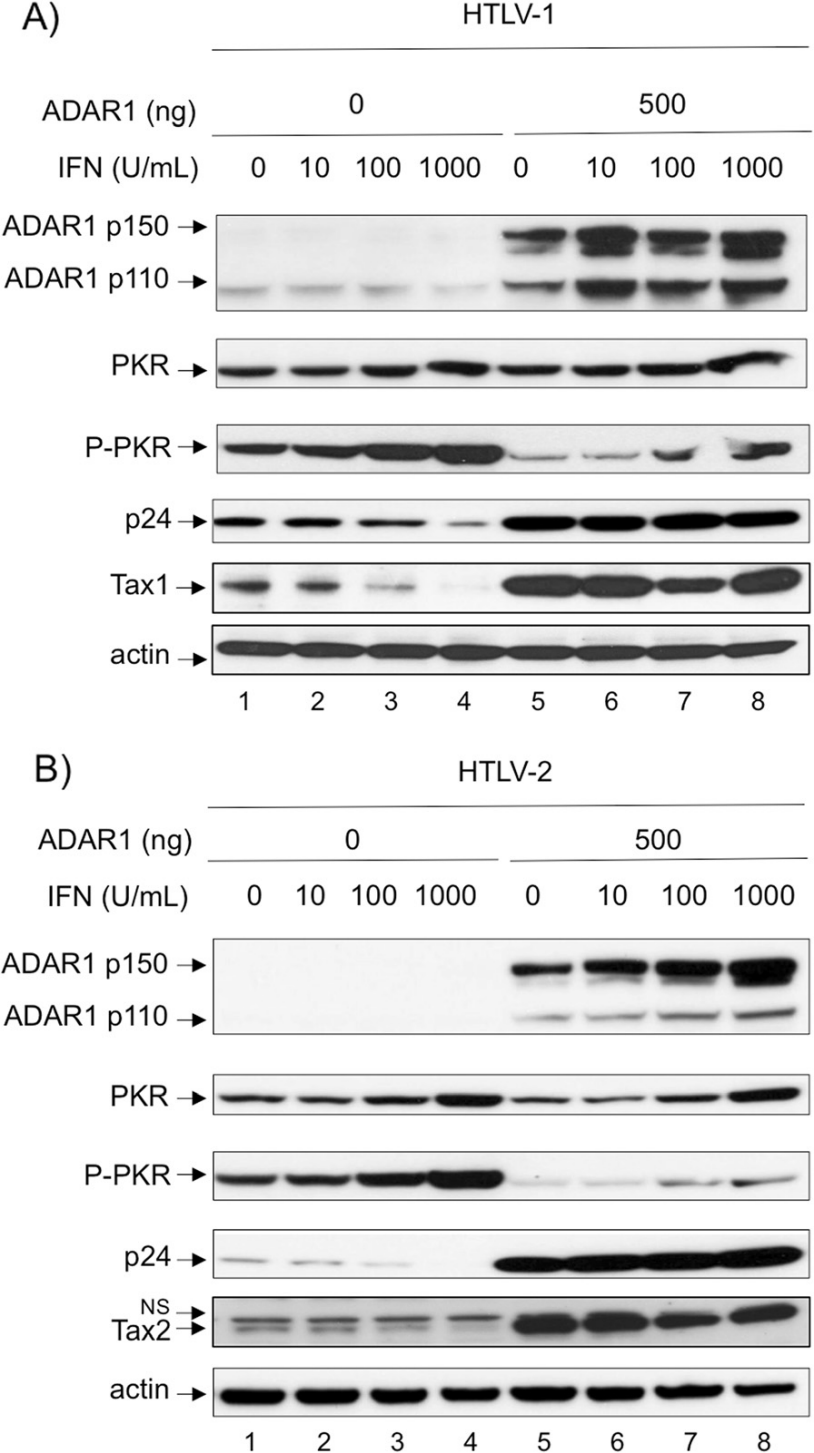
**ADAR1 expression reverses IFN-** ***α*** **inhibitory effect on HTLV-1 and HTLV-2.** 293--T cells were transfected with 4 μg of **(A)** pACH or **(B)** pH6neo molecular clone. When required, 500 ng of an ADAR1 expression plasmid were also transfected and cells were treated with increasing amount of IFN-*α* (0-1000 U/mL) for 48 hours. Western blot analyses were performed on 60 μg of proteins obtained from whole cell extracts using anti-ADAR1, anti-p24^gag^, anti-Tax1, anti-Tax2, anti-PKR, anti-phospho-PKR and anti-actin antibodies.

293-T cells were transfected with HTLV-1 (Figure 7A) or HTLV-2 molecular clones (Figure 7B), with or without an ADAR1 expression plasmid, before being treated with increasing amounts of IFN-α. In the absence of ADAR1 over-expression and consistent with our previous data [11], IFN-α treatment led to a decreased p24^gag^ as well as Tax1 or Tax2 protein expression (Figure 7A and B, compare lane 1 and 4). This effect was correlated with an increase in PKR and phospho-PKR levels. In the presence of ectopic ADAR1, IFN-α treatment still lead to increased PKR levels, but the levels of phospho-PKR remained low and were not associated with a decreased p24^gag^ or Tax expression (Figure 7A and B, compare lane 5 and 6-8).

These results show that IFN-α cannot activate PKR in the presence of elevated levels of ADAR1, thus ADAR1 expression prevents PKR from having its antiviral role.

## Discussion

ADAR1 is responsible for A-to-G (I) editing of cellular and viral genes [30,31]. Editing can be site-selective in the case of Hepatitis virus D [41,42] or of L-glutamate receptor [60]. It can also be non-selective in the case of Hepatitis C, influenza, measles and Rift valley viruses [31,33,44]. As ADAR1 expression is induced by IFN-I, it was originally anticipated that IFN-I antiviral effects occur at least partly through an ADAR1 dependent editing of viral genomes. Indeed, Taylor *et al*. demonstrated that IFN-α treatment induces A-to-I editing in HCV replicon cells, while HCV editing is reduced when ADAR1 enzymatic activity is inhibited [33].

We previously demonstrated that co-transfecting ADAR1 together with HTLV-2 or STLV-3 molecular clones in 293-T cells led to A-to-G editing although the frequency of such editing could not be quantified [57]. A similar observation was made for measles and influenza viruses [44]. However, such hyper-edited sequences were only detected using the exquisitely sensitive 3DI-PCR technique [31], and replication of those viruses was not tested. In addition, editing was not observed in a series of samples obtained from 20 HTLV-2 infected individuals, although ADAR1 mRNA was present in those samples. This suggested that A-to-G (I) editing is an extremely rare phenomenon *in vivo*, at least in HTLV-2 infected cells [57].

As HTLV-1 mostly infects CD4^+^ T-lymphocytes and HTLV-2 infects CD8^+^ T-lymphocytes [16], we measured here ADAR1 p110 and p150 expression in peripheral blood lymphocytes that were left quiescent or PHA/IL-2 activated. Consistent with a previous study [61], both ADAR1 isoforms were present in activated lymphocytes. Thus, ADAR1 is expressed in cells that are targets of HTLV-1 and HTLV-2. Consistent with data obtained on HTLV-2 *ex vivo* samples [57], we have also shown that ADAR1 expression is higher in HTLV-1 and HTLV-2 chronically infected lymphocytes compared to non-infected T-cells and in primary CD4^+^ T-cells obtained from HTLV-1 infected individuals, thus suggesting that ADAR1 is expressed in response to HTLV infection. Interestingly, Clerzius *et al* have also shown that ADAR1-p110 and ADAR1-p150 expression is increased when Jurkat cells are infected with HIV-1 [50]. It could be of interest to determine in the future which factor induces ADAR1 expression in HTLV-1/-2 infected cells.

ADAR1 proviral effect was previously observed for different viruses [36,37,40,49-52]. This effect is mediated through (1) viral RNA editing, (2) the inhibition of PKR through the formation of an ADAR1/PKR complex [37,50] or (3) an increase in ADAR1 catalytic activity. We have shown here that ADAR1 effect on HTLV-1/-2 replication is independent from ADAR1 editing activity and is rather linked to the inactivation of PKR function. It could also be of interest to determine whether ADAR1 effect is also linked to the formation of a complex between those two cellular proteins. Of note, our results do not exclude the fact that ADAR1 editing on HTLV-1/2 genomes might also occur, but at a very low frequency thus having no deleterious effect on viral cycle.

Shoggins *et al* have recently developed an assay to study the effect of hundreds of ISG products on different viruses that belong to *Flaviviridae* (YFV, WNV), *Togaviridae* (CHIKV, VEEV) or *Retroviridae* (HIV-1) [53]. This led to the identification of ISGs that are strong repressors, modest inhibitors, but also that are activators of viral cycle. In this study, ADAR was identified as an enhancer of replication for all viruses tested. Interestingly, the authors also tested the combination of one inhibitory and one enhancing ISG on viral cycle, and showed that, in most cases, viral replication was more influenced by the inhibitory than by the enhancing ISG [53]. Thus, this study highlighted that IFN-I does not necessarily promote an antiviral state and that a subtle balance between repressor and activator ISGs regulate the fate of viral infections.

We have previously shown that IFN-α inhibits HTLV-1 and HTLV-2 *de novo* infection through PKR activation [11]. We demonstrated here that another ISG, ADAR1 p150 is expressed in activated T-cells and plays a proviral role on HTLV-1 and HTLV-2 replication. We can therefore elaborate a working hypothesis that, during primary infection and in the absence of IFN-I, HTLV-1/-2 will infect cells that express ADAR1 p150 but not PKR. In this case, ADAR1 will play a proviral role. Infection could then lead to IFN-I production that could in turn activate both PKR and ADAR-1 p150 in surrounding cells. In this case, according to Schoggins *et al*., PKR could play an antiviral role [53]. Given that IFN-I is used to treat chronic ATL patients [62], it would therefore be interesting to measure quantitatively the expression of both PKR and ADAR in ATL cells if those samples were available.

## Conclusions

In conclusion, our results demonstrate that IFN-I promotes the expression of two ISG that have antagonist roles on HTLV replication.

## Methods

### Cell culture and flow cytometry

293T and 293T-LTR-GFP cells were maintained in Dulbecco’s modified Eagle’s medium (DMEM) supplemented with 10% fetal bovine serum (Gibco, Life technologies) and 100 μg/mL penicillin-streptomycin (Gibco, Life technologies). Jurkat, Jurkat-LTR-luc, HTLV-1- (C91/PL, MT2) and HTLV-2-infected T-cell lines (C19, MO), were maintained in RPMI 1640 medium supplemented with 10% fetal bovine serum (Gibco, Life technologies) and 100 μg/mL penicillin-streptomycin (Gibco, Life technologies). Peripheral blood lymphocytes (PBL) were purified from blood samples obtained from healthy donors (Etablissement Français du Sang, Lyon) or from HTLV-1 infected individuals. HTLV-1 infected blood samples were obtained in the context of a Biomedical Research Program approved by the "comité de protection des personnes", Ile de France III, Paris (2007-A01103-50, number 2494) with informed consent of all individuals. Cells maintained in RPMI 1640 medium supplemented with 10% fetal bovine serum (Gibco, Life technologies) and 100 μg/mL penicillin-streptomycin (Gibco, Life technologies). PBL were either left quiescent or stimulated with phytohemagglutinin (PHA, 1 μg/mL, SIGMA) and interleukin-2 (IL-2 at 150 U/mL). When required, IFNα-2a (Tebu-Bio) was added for 3 days to the cells. 293T-LTR-GFP and Jurkat-LTR-luc are stably transfected with a plasmid encoding GFP (green florescent protein) or luciferase under the control of HTLV-1 long terminal repeat (LTR), respectively [63,64]. All cell lines were grown at 37°C in 5% CO_2_.

### B-cells analyses

CD19^+^ B cells were purified by depletion/negative selection using a B-cell isolation kit (Miltenyi Biotech). Purity of isolated B-cells was monitored using anti-hCD19 VioBlue (Miltenyi Biotech) and was analyzed by fluorescence-activated cell sorting (FACS CantoII; BD Biosciences).

B-lymphocytes were cultured in RPMI 1640 medium (Gibco BRL Invitrogen) supplemented with 10% FCS and 1% penicillin/streptomycin with 50 ng/ml IL2 (Miltenyi Biotech). They were either treated with Pansorbin 200 ng/ml (Millipore Calbiochem) (activated condition) or left untreated (non activated condition). 24 h and 48 h later cells were labeled with hCD19-PE, hCD3-PE and hCD69-PEvio770 (Miltenyi Biotech). ADAR antibodies (Abcam) were used after permeabilization/fixation using the Cytofix/Cytoperm kit (BD).

### T-cells analyses

CD4^+^ T-cells obtained from healthy donors or HTLV-1 infected individuals were purified by depletion/negative selection using T-cell isolation kit (Miltenyi Biotech). Purity of CD4^+^ T-cells was monitored using anti-hCD4APC antibodies (Miltenyi Biotech) and was analyzed by fluorescence-activated cell sorting (FACS CantoII; BD Biosciences). CD4^+^ T-lymphocytes were then cultured at 1.10^6^/ml with IL-2 (150 U/ml) and PHA (1 μg/ml) for 3 days and then maintained only with IL-2 for 5 days. Cells were analyzed with a surface staining against hCD4-APC and hCD69-PE (Miltenyi Biotech) or intracellular staining for ADAR and p19 (Zeptometrix) after permeabilization/ fixation with the Cytofix/Cytoperm kit (BD). Cells were analyzed on a FACS-Canto II (BD Sciences) collecting 100.000 events. Results were analyzed using the FlowJo software.

### Plasmids

HTLV-1 proviral DNA clone (pACH) was provided by Dr L. Ratner [65]. HTLV-2 proviral DNA clone (pH6neo) and SV2Neo plasmids were provided by Dr P. Green [66]. pSG5M-Tax1, pSG5M-Tax2, HTLV-1- and HTLV-2-LTR-luciferase plasmids were previously described [15,67]. ADAR1 wild type and editing-deficient (HAE - > HAG mutation at the position 912) [68] were provided by Dr P. Auewarakul [52]. The 3XF-ADAR1 RNA editing reporter system was provided by Dr. V. Lotteau [40]. PKR wild-type plasmid was provided by Dr E. Meurs [69]. Optineurin wild-type plasmid was previously described [70].

### Cell-to-cell infection experiments

Jurkat (10^6^) or PBLs (5.10^5^) were transfected with 2.5 μg of the HTLV-1 or the HTLV-2-LTR-luciferase plasmids and 2.5 μg of the ADAR1 plasmid (Neon® Transfection System, Invitrogen) following the manufacturer’s instructions. Twenty-four hours later, they were co-cultured with irradiated (77 Gy CIS, BIO international, IBL 637) C91-PL or C19 cells (3:1 ratio). When needed, PBLs were treated with 50 μM AZT (Sigma) 24 hours and 3 hours before infection. Twenty-four hours later, reporter activity was assayed (Luciferase reporter assay system, Promega). Luciferase activity was normalized by protein concentration as determined by the Bradford method (Bio-Rad).

293T (3.10^6^) cells were seeded onto 100 mm dishes. Twenty-four hours later, cells were transfected with 4 μg of HTLV-1 (pACH) or HTLV-2 (pH6neo) molecular clones and increasing amount (0, 5, 50, 500 ng) of ADAR1 expression plasmid (Polyfect, Qiagen). Forty-height hours post-transfection, 293 T cells were washed and co-cultured with reporter Jurkat-LTR-Luciferase (1:3 ratio) cells for 24 hours. Reporter activities were assayed using the luciferase reporter assay system (Promega) and normalized by protein concentration as determined by the Bradford method (Bio-Rad).

### Transfections with HTLV molecular clones

293T cells (3.10^6^) were seeded onto 100 mm dishes. The following day, 4 μg of pACH (HTLV-1), pH6neo (HTLV-2) or SV2Neo (control) plasmids were transfected with or without 5, 50, 500 ng of ADAR1 using PolyFect reagent (Qiagen) following manufacturer's instructions and cells were treated with increasing amount (0, 10, 100, 1000 U/mL) of IFN-α2a (Tebu-Bio) for 48 hours. Reporter activity was assayed (Luciferase reporter assay system, Promega) and normalized by protein concentration as determined by the Bradford method (Bio-Rad).

Jurkat cells (10^6^) or PBL (5. 10^5^) were transfected with 2 μg of the HTLV-1 or the HTLV-2-LTR-luciferase plasmids, 2 μg of the HTLV-1 (pACH) or HTLV-2 (pH6neo) molecular clones with or without 1 μg of the ADAR1 expression plasmid (Neon® Transfection System, Invitrogen) following manufacturer’s instructions. Forty-eight hours later reporter activity was assayed (Luciferase reporter assay system, Promega). Luciferase activity was normalized by protein concentration as determined by the Bradford method (Bio-Rad).

### Fluorescence microscopy

293T-LTR-GFP cells were seeded at 5. 10^5^ cells/well on 6-well plates. The next day, one microgram of the appropriate plasmid was transfected as described above, with or without the ADAR plasmid. Two days later, cells were visualized and pictures were captured with a Zeiss Axio Vert.A1 imaging microscope.

### p19^gag^ ELISA

Cell culture supernatants were collected 48 hours post-transfection and the presence of the HTLV matrix p19^gag^ protein was quantified using a commercially available ELISA kit (Retrotek HTLV-1/2 p19 antigen, Zeptometrix) following the manufacturer’s instructions as previously described [11]. In brief, wells are coated with polyclonal antibodies which react with the major gag gene products of HTLV-1 and HTLV-2. Viral antigen in the test specimen is captured by the antibody.

### PKR silencing

293T cells were seeded at a concentration of 3.10^5^ cells per well onto 6-well plates. The following day, 20 nM of PKR siRNA (ON-TARGETplus SMART pool EIF2AK2, Fermentas) or control siRNA (ON-TARGETplus Non-targeting Pool, Fermentas) were transfected (HiPerfect reagent, Qiagen) as previously described [11]. Twelve hours post-transfection, 1.2 μg of pACH or PH6neo plasmid were transfected together with 20 nM of siRNA and 125 ng of ADAR1 (Attracten reagent, Qiagen) following the manufacturer's instructions. Cells were collected 48 hours later and analyzed.

### Tax Ni-NTA immunoprecipitation

293T cells were seeded in 10-cm dishes and transfected (Polyfect, Qiagen) with a Tax1-His-encoding plasmid (4 μg), together with an OPTN- or a PKR-encoding plasmid (4 μg). Twenty-four hours later, cells were harvested and lysed in Chris buffer (50 mM Tris, 0.5% Nonidet P-40, 200 mM NaCl, 0.1 mM EDTA, 100 mM NaF, 2 mM Na_3_VO_4_, 10 mM imidazole) in the presence of protease inhibitors (Complete, Roche Diagnostics) and incubated with Ni^2+^-NTA (nitrilotriacetic acid) beads (Sigma) at +4°C under agitation. Beads were then washed extensively in Chris buffer. Bound proteins were eluted in Laemmli buffer and processed for immunoblot analyses. The following antibodies were used: anti-His (sc-804, Santa Cruz), anti-OPTN (#100 000, Cayman Chemical), anti-PKR (71/10, [71]).

### Immunoblot analyses

Cells were washed with PBS, lysed (50 nM Tris-HCl pH 7.4, 150 nM NaCl, 5 mM EDTA, 0.5% Nonidet-P-40, 0.2 mM Na3VO4, 50 mM NaF, 1 mM dithitheitol, 1 mM phenylmethylsulfonyl fluoride) and protease inhibitors (Complete Roche Applied Science) as previously described [67]. Cell debris were pelleted by centrifugation. Protein concentration was determined by the Bradford method (Bio-Rad). Sixty micrograms of proteins were loaded onto 4-12% NUPAGE gels (NOVEX, Invitrogen), subjected to electrophoresis at 150 V, and transferred onto a PVDF membrane (Immobilon-P, Millipore). Membranes were blocked in a 5% milk-PBS-0.05% Tween 20 solution, then incubated overnight with the primary antibody (anti-ADAR1 (Abcam ab88574, 1:1000), anti-PKR 71/10 (1:500) [71], anti-phospho PKR (Epitomics #1120-1, 1:2000), anti-Tax-1-specific (Tab 172, 1:1000), anti-Tax-2 (GP3738, 1:4000), [17] anti-HTLV-1/2 p24 (Zeptometrix 75/4.21.11, 1:400), anti-β-actin clone AC74 (Sigma, 1:40000). The next day, membranes were washed and incubated either with anti-rabbit or with anti-mouse horseradish peroxidase-conjugated secondary antibodies and developed using ECL Plus reagent kit (GE Healthcare).

## Additional files

Additional file 1: Figure S1. Testing the editing capacity of ADAR1wt and ADAR1 mutant constructs. 293-T cells were transfected with 150 ng of the 3XF-ADAR1 RNA editing reporter system with (left) 0, 150, 300, 600, 1000 ng of the plasmid coding for ADAR1 wild-type or with (right) 0, 150, 300, 600, 1000 ng of the plasmid encoding ADAR1 with a mutation in its catalytic site. Forty-eight hours later, luciferase activity was measured and results normalized to renilla expression and calculated as fold change compared to cells non transfected with ADAR1 plasmid arbitrarily set to 100%. Data are the mean ± standard deviation (SD) from 2 independent experiments. Asterisks indicate statistically significant differences between treated and untreated cells (paired Student *t* test, *p < 0.05; NS: non significant). Additional file 2: Figure S2. Viral particles produced in the presence of an editing-deficient ADAR1 are still infectious. 293-T cells were transfected with 4 μg of (A) ACH or (B) pH6neo molecular clone together with 500 ng of wild type or mutant ADAR1. Forty-eight hours later, cells were washed and co-cultured with Jurkat-LTR-luc cells for 24 hours (ratio 1:3). Luciferase activity was normalized by protein concentration as determined by the Bradford method and calculated as fold change compared to cells non transfected with ADAR1 plasmid arbitrarily set to 100%. Paired Student *t* test, NS: not significant.
