# Supplementary figures and images for: ADAR1 enhances HTLV-1 and HTLV-2 replication through inhibition of PKR activity
Source: Retrovirology. 2014 Nov 12;11:93. doi: 10.1186/s12977-014-0093-9 (PMC4245799; doi:10.1186/s12977-014-0093-9)

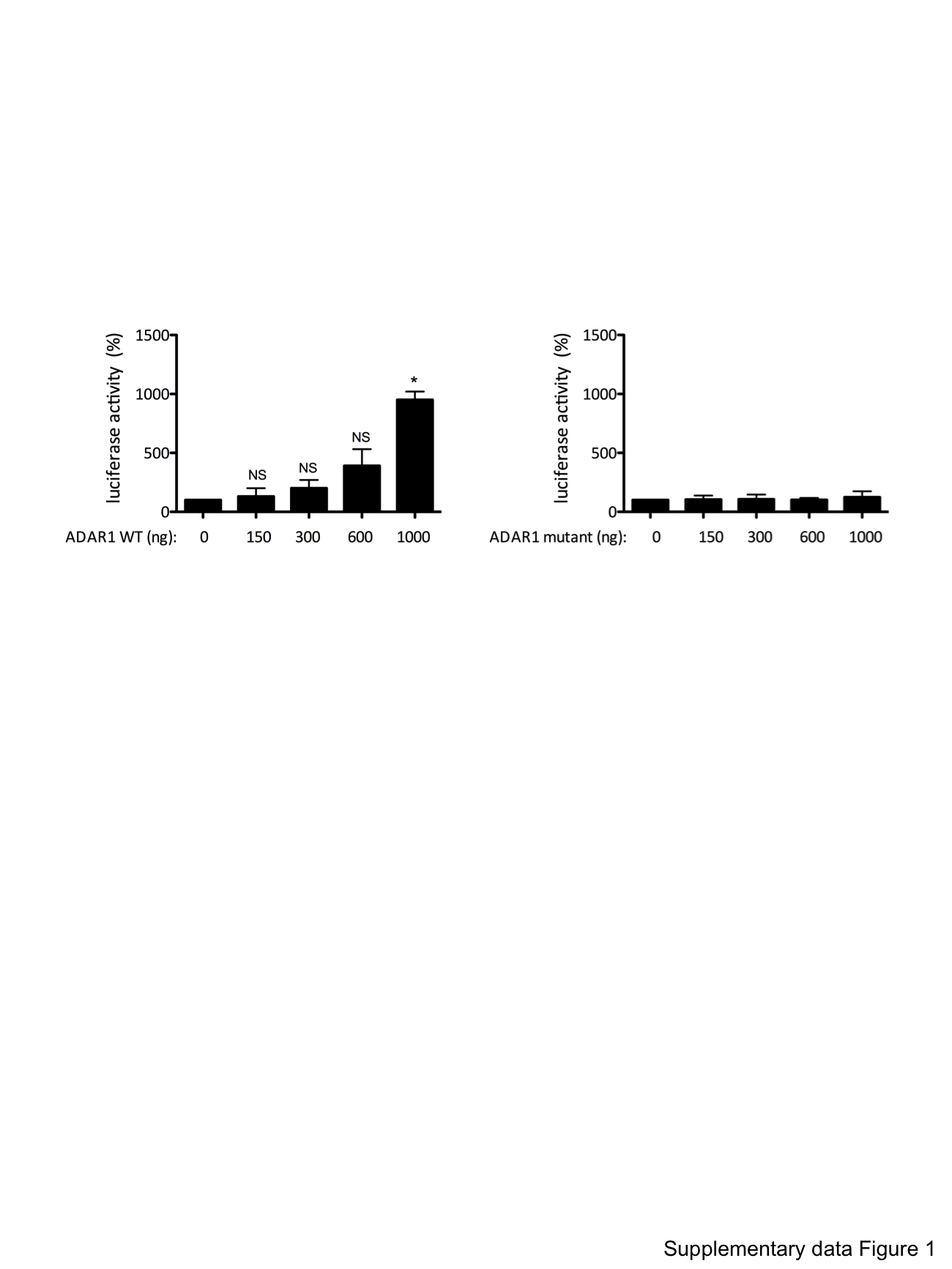

Supplement: Additional file 1: Figure S1. — Testing the editing capacity of ADAR1wt and ADAR1 mutant constructs. 293-T cells were transfected with 150 ng of the 3XF-ADAR1 RNA editing reporter system with (left) 0, 150, 300, 600, 1000 ng of the plasmid coding for ADAR1 wild-type or with (right) 0, 150, 300, 600, 1000 ng of the plasmid encoding ADAR1 with a mutation in its catalytic site. Forty-eight hours later, luciferase activity was measured and results normalized to renilla expression and calculated as fold change compared to cells non transfected with ADAR1 plasmid arbitrarily set to 100%. Data are the mean ± standard deviation (SD) from 2 independent experiments. Asterisks indicate statistically significant differences between treated and untreated cells (paired Student t test, *p < 0.05; NS: non significant). [file 12977_2014_93_MOESM1_ESM.png]

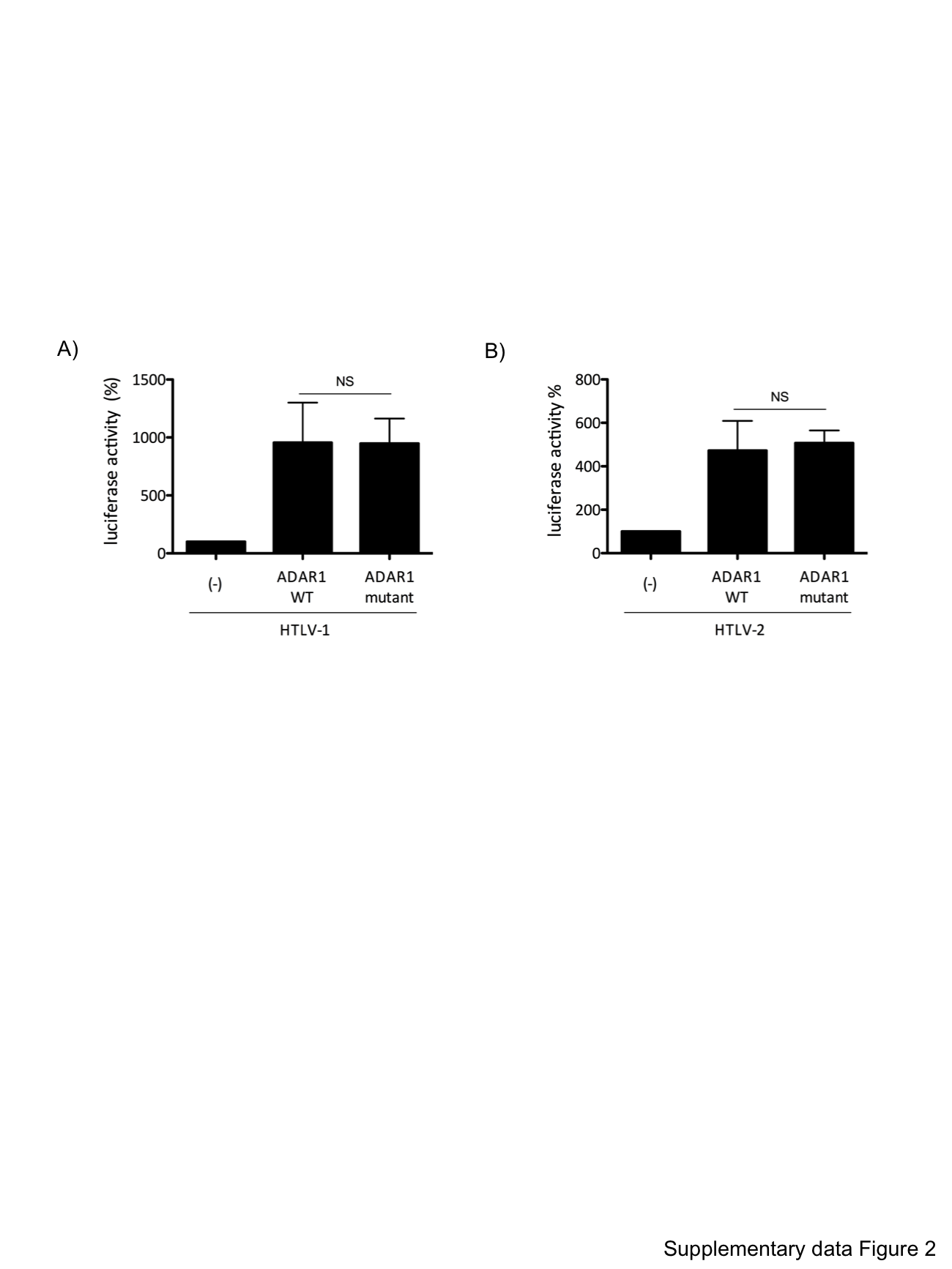

Supplement: Additional file 2: Figure S2. — Viral particles produced in the presence of an editing-deficient ADAR1 are still infectious. 293-T cells were transfected with 4 μg of (A) ACH or (B) pH6neo molecular clone together with 500 ng of wild type or mutant ADAR1. Forty-eight hours later, cells were washed and co-cultured with Jurkat-LTR-luc cells for 24 hours (ratio 1:3). Luciferase activity was normalized by protein concentration as determined by the Bradford method and calculated as fold change compared to cells non transfected with ADAR1 plasmid arbitrarily set to 100%. Paired Student t test, NS: not significant. [file 12977_2014_93_MOESM2_ESM.png]
